# Dietary Supplement and Food Contaminations and Their Implications for Doping Controls

**DOI:** 10.3390/foods9081012

**Published:** 2020-07-27

**Authors:** Katja Walpurgis, Andreas Thomas, Hans Geyer, Ute Mareck, Mario Thevis

**Affiliations:** 1 Center for Preventive Doping Research/Institute of Biochemistry, German Sport University Cologne, 50933 Cologne, Germany; a.thomas@biochem.dshs-koeln.de (A.T.); h.geyer@biochem.dshs-koeln.de (H.G.); u.mareck@biochem.dshs-koeln.de (U.M.); thevis@dshs-koeln.de (M.T.); 2 European Monitoring Center for Emerging Doping Agents (EuMoCEDA), 50933 Cologne/Bonn, Germany

**Keywords:** doping, sport, contamination, SARMs, diuretics

## Abstract

A narrative review with an overall aim of indicating the current state of knowledge and the relevance concerning food and supplement contamination and/or adulteration with doping agents and the respective implications for sports drug testing is presented. The identification of a doping agent (or its metabolite) in sports drug testing samples constitutes a violation of the anti-doping rules defined by the World Anti-Doping Agency. Reasons for such Adverse Analytical Findings (AAFs) include the intentional misuse of performance-enhancing/banned drugs; however, also the scenario of inadvertent administrations of doping agents was proven in the past, caused by, amongst others, the ingestion of contaminated dietary supplements, drugs, or food. Even though controversial positions concerning the effectiveness of dietary supplements in healthy subjects exist, they are frequently used by athletes, anticipating positive effects on health, recovery, and performance. However, most supplement users are unaware of the fact that the administration of such products can be associated with unforeseeable health risks and AAFs in sports. In particular anabolic androgenic steroids (AAS) and stimulants have been frequently found as undeclared ingredients of dietary supplements, either as a result of cross-contaminations due to substandard manufacturing practices and missing quality controls or an intentional admixture to increase the effectiveness of the preparations. Cross-contaminations were also found to affect therapeutic drug preparations. While the sensitivity of assays employed to test pharmaceuticals for impurities is in accordance with good manufacturing practice guidelines allowing to exclude any physiological effects, minute trace amounts of contaminating compounds can still result in positive doping tests. In addition, food was found to be a potential source of unintentional doping, the most prominent example being meat tainted with the anabolic agent clenbuterol. The athletes’ compliance with anti-doping rules is frequently tested by routine doping controls. Different measures including offers of topical information and education of the athletes as well as the maintenance of databases summarizing low- or high-risk supplements are important cornerstones in preventing unintentional anti-doping rule violations. Further, the collection of additional analytical data has been shown to allow for supporting result management processes.

## 1. Introduction

According to the World Anti-Doping Code (WADC), doping is defined as a violation of the Anti-Doping Rules [1], comprising, *inter alia*, the detection of a prohibited substance, its metabolites, or markers in the blood or urine sample of an athlete. However, there are different scenarios where such an Adverse Analytical Finding (AAF) does not necessarily result from a deliberate application of a performance-enhancing/banned drug (*vide infra*). Such cases of inadvertent doping include the ingestion of adulterated or faked dietary supplements, tainted food, and contaminated drugs, as well as passive exposure to doping agents or an insufficient education of the athletes with regards to changes of the Prohibited List annually revised by the World Anti-Doping Agency (WADA) [2,3,4,5,6]. According to WADA’s policy of strict liability, an athlete is responsible for the substances found in his/her doping control samples and anti-doping rule violations (ADRVs) occur regardless of his/her intention [1,7]. Possible consequences comprise not only temporary or permanent suspensions, but also loss of medals and/or records, financial sanctions, damage to the athlete’s reputation, and failed sponsorships [3,8]. However, the decision-making processes are flexible to consider the circumstances, so that clear evidence about the origin of the detected prohibited substance can potentially lead to reduced sanctions [1,4,7]. On the other hand, it cannot be excluded that athletes occasionally argue with contamination scenarios in an attempt to excuse an AAF in order to avoid impending penalties [2,5]. Consequently, a careful interpretation of the results and, if available, additional data (e.g., from microdose elimination studies) are necessary and desirable.

WADA statistics of the years 2013–2017 demonstrated that between 4 and 19% of the reported AAFs were not sanctioned due to an exoneration of the athlete [9,10,11,12,13]. Reasons included, amongst others, dietary supplement or meat contaminations. In this narrative review, suspected and proven incidences of food and supplement contamination and/or adulteration with doping agents and the respective implications for sports drug testing are presented and discussed. Analytical approaches employed in anti-doping research and routine analysis concerning the presented investigations into presumed contamination scenarios are exclusively based on chromatographic-mass spectrometric methods, offering specificity and sensitivity for conclusive result interpretation. The discussion includes both theoretical and contextual points of view, with an overall aim of indicating the current state of knowledge and the relevance and need for future research into specific areas.

## 2. Dietary Supplements

### 2.1. Overview

Since ancient times, athletes try to improve their strength, speed, agility, and bravery by using special diets and products such as lion hearts and deer livers [6,14]. With the growing scientific understanding of exercise physiology in the early 20th century, more specialized dietary supplements and ergogenic aids were employed to increase physical fitness [14].

In general, athletic performance depends on a variety of factors such as talent, motivation, training, and the resistance to injuries, but the individual potential can be optimized by a healthy and appropriate diet [8,15,16]. An additional application of dietary supplements can be reasonable for athletes with nutritional challenges (e.g., vegans) or in certain medical circumstances (e.g., a diagnosed nutrient deficiency); however, for many of them, health and performance enhancing effects are not proven [6,8,15,17,18]. Therefore, they should only be used after consultation of a physician or sports nutritionist [8,15]. Nevertheless, supplement use is nowadays widespread among athletes at all levels of sport, especially as they are readily available without medical prescription [8,18]. According to data obtained from doping controls during the Olympic Games held in Sydney and Athens in 2000 and 2004 [19,20], 78% and 75.7% of the tested athletes used dietary supplements and/or medications during the last three days before testing. The evaluation of 3887 doping control forms collected by the International Association of Athletics Federations (IAAF) both in- and out-of-competition between 2003 and 2008 yielded an average use of 1.7 supplements and 0.8 medications per athlete within the preceding 7 days [21]. Further, during the FIFA World Cups 2002 and 2006, the physicians of the participating teams reported a usage of 1.8 substances per player and match, of which 57.1% were dietary supplements and 42.9% were medications [22]. In 2009, Braun et al. published the results of a questionnaire which was conducted to assess the prevalence of supplement use among 164 young German elite athletes [23]. A total of 80% of the study participants declared the past or present use of at least one supplement, and a significant difference was observed between age groups (older > younger athletes) and performance-levels (in some countries referred to as A/B-level > C/D-level). In addition, in 2019 Baltazar-Martins et al. [24] reported the use of dietary supplements by 64% of 527 surveyed elite athletes.

The reasons for resorting to such aids are manifold: To generally improve health and prevent or cure illnesses/injuries, to promote recovery from training, to directly or indirectly increase athletic performance, to treat a presumed nutrient deficiency due to an unbalanced diet, for weight loss, to enhance mood, or to conveniently provide nutrients and energy when required [6,8,15,17,18,23].

In a recently published consensus statement, dietary supplements are defined as the following: A food, food component, nutrient, or non-food compound that is purposefully ingested in addition to the habitually consumed diet with the aim of achieving a specific health and/or performance benefit. [8]. They comprise sports foods (e.g., sports drinks/bars/gels, protein powders), single nutrients with minerals or vitamins, and ergogenic aids (e.g., caffeine, creatine) as well as superfoods (e.g., chia seeds, goji berry extracts), herbal/botanical products, foods enriched with certain ingredients (e.g., vitamin-/mineral-fortified), and multi-ingredient preparations [8,17].

Even though controversial opinions exist concerning the general effectiveness of dietary supplements in healthy subjects, some products might be beneficial for certain types of athletes when used in appropriate dosing and administration schemes [8,15]. For example, products offering concentrated protein and amino acid supply represent convenient options for strength and power athletes to achieve the necessary level of protein intake without a concurrent fat load [14,15]. Creatine is an organic compound endogenously synthesized from amino acids, which is transported into the muscle and enzymatically converted to creatine phosphate. This, in turn, represents an important source of energy under anaerobic conditions and partially restores muscle ATP content during recovery [8,15,25,26]. Therefore, an additional creatine supplementation is supposed to be favorable especially in strength and team sports involving intermittent high-intensity exercise. Alkalizing agents such as sodium bicarbonate and beta alanine can increase the buffering capacity in muscles when the pH is a limiting factor due to anaerobic glycolysis and a rapid breakdown of glycogen to lactate [8,15,16]. Dietary nitrate improves the bioavailability of nitric oxide (NO), which is an important modulator of skeletal muscle function [8]. The intake of chondroitin and glucosamine, representing main constituents of cartilage, have been mentioned as potentially instrumental in improving joint cartilage conditions of athletes [15], and lastly caffeine, which is a stimulant currently not prohibited in sports, and has been shown to support both physical and mental performance in selected studies [8,15,16].

### 2.2. Risks Associated with the Use of Dietary Supplements

Athletes using dietary supplements are not only susceptible to acute or long-term damage to their health but also to inadvertent doping [8,15]. While the safety, purity, and efficacy of pharmaceutical products are thoroughly and continuously controlled, no uniform regulations and quality controls exist for the manufacturing of dietary supplements, resulting in a highly variable quality of the available preparations [2,15,27,28,29,30].

The main problem for the general population and especially for athletes is an inaccurate labelling of ingredients, which is of concern to all types of dietary supplements including pills, powders, capsules, and liquids [2,8,15,27,28,31,32,33]. While especially those products featuring comparably expensive components occasionally contain only little (if any) active ingredient [15,27], dietary supplements cross-contaminated or even intentionally fortified with undeclared performance-enhancing substances such as anabolic agents or stimulants in order to increase their efficacy are significantly more worrying [2,8,28,31,34]. Moreover, the use of varying (chemical) synonyms of prohibited substances on product labels adds another level of complexity for athletes to recognize a potential issue [2,8,32].

Cross-contaminations are commonly the result of one of two scenarios: Either inappropriately cleaned containers are used for the transportation or storage of the raw materials or dietary supplements, especially when other preparations such as prohormones are manufactured in the same production line [15,28,31,34,35,36]. Even though selected reputable manufacturers, working according to Good Manufacturing Practicing (GMP) regulations, have identified risk factors and installed quality controls accordingly, the situation is further complicated by the fact that the source of some cross-contaminations is not necessarily the facility, where the final products are manufactured [35]. Therefore, product and/or raw material testing needs to be conducted with assays that are applicable to all types of relevant matrices and have limits of detection (LODs) in the low ng/g or parts per billion (ppb) range. Such sensitivities are necessary to account for the excellent detection limits of currently employed analytical methods in sports drug testing and the facts that for many substances any detected amount constitutes an AAF in routine doping controls with some dietary supplements being administered in relatively large amounts [6,31,34,35]. Moreover, batch-to-batch, package-to-package, and even tablet-to-tablet variations can occur.

Even if the resulting concentrations of a prohibited drug are too low to have any physiological effect, they can cause an AAF in sports [8,31,34] Therefore, athletes are advised to use available sources to identify “low-risk” products and prevent unintentional ADRVs due to the administration of contaminated/adulterated dietary supplements [28]. In some countries such as Germany and The Netherlands, athletes can obtain such information from databases cataloguing only tested products from manufacturers performing quality controls on a regular basis, either in-house or by using third-party companies as e.g., analytical laboratories [6,15,28,31,36,37]. Moreover, some anti-doping organizations as for example the US Anti-Doping Agency (USADA) have listed high-risk dietary supplements on a dedicated website [32,38].

#### 2.2.1. Anabolic Agents

Since decades, anabolic agents promising positive effects on muscle mass, strength, and recovery, are the drugs most frequently detected in doping control samples [39]. Their usage is prohibited both in- and out-of-competition and, according to current WADA statistics [40], 44% of the AAFs reported in 2018 were anabolic agents. Besides exogenous anabolic androgenic steroids (AAS) as for example metandienone and stanozolol, this substance class includes also endogenous AAS of exogenous origin such as testosterone and nandrolone, and other anabolic agents as for instance selective androgen-receptor modulators (SARMs) and clenbuterol [36,39,41]. While exogenous AAS are routinely detected in biological samples employing gas chromatography-mass spectrometry (GC-MS) or liquid chromatography-mass spectrometry (LC-MS), abnormal steroid/metabolite concentrations and/or ratios within the steroidal module of the athlete biological passport (ABP) and isotope-ratio mass spectrometry (IRMS) are required to provide evidence for the misuse of endogenous AAS [36,39].

Over the last years, numerous dietary supplements were found to be cross-contaminated with different prohormones or unlabeled AAS such as stanozolol, metandienone, boldenone, and oxandrolone [28].

Prohormones of AAS including dehydroepiandrosterone (DHEA), 4-androstenedione, 4-androstenediol, 5-androstenediol, and different 19-norsteroids are sold as dietary supplements with anabolic properties in the US and several other countries for more than 20 years [36,42,43]. Following ingestion, they are enzymatically converted to testosterone and nandrolone, and therefore also included in the WADA Prohibited List [41]. The misuse of testosterone and its prohormones in sports can be corroborated by an elevated testosterone/epitestosterone ratio (T/E) or abnormal metabolite concentrations/ratios within the steroidal module of the ABP as well as IRMS [36,43,44,45]. By contrast, the administration of nandrolone and the corresponding prohormones lead to the detection of the urinary metabolite 19-norandrosterone, whose exogenous origin has to be additionally confirmed by means of IRMS if the urinary concentration ranges between 2.5 and 15 ng/mL [43,46]. As many manufacturers of prohormones also produce other non-hormonal dietary supplements, inadequate manufacturing practices and substandard quality controls can result in contaminated products and inadvertent doping in sports [42].

The first cases of dietary supplements contaminated with AAS were reported in 2000 in the context of several AAFs with norandrosterone [43]. The affected athletes used products labeled to contain the flavonoids chrysin/quercetine or plant-derived ingredients attributed to *tribulus terrestris* and *guarana*. However, following extraction, derivatization, and GC-MS analysis, different prohormones of testosterone and nandrolone were identified in these products. As high batch-to-batch and capsule-to-capsule variations were observed and the detected total amounts of 0.3–5100 µg/capsule were significantly lower than in commercially available prohormone preparations (~25 mg per capsule), cross-contaminations appeared more likely than an intentional admixture. Nevertheless, an administration study demonstrated that the ingestion of one capsule only of each of the analyzed products can lead to positive findings with the nandrolone metabolites 19-norandrosterone and 19-noretiocholanolone. Also, the T/E of a female study participant was found elevated.

In the same year, GC-MS analysis of a US supplement labeled to contain different plant extracts, L-carnitine, phenylalanine, vitamin B_6_, and other ingredients irrelevant in a doping control context, revealed the presence of the testosterone prohormone 4-androstenedione (0.7 mg/capsule) and the nandrolone precursor 19-norandrostenedione (4.8 mg/capsule) [47]. While the administration of one capsule to five healthy volunteers did not change the T/E or androstenedione/E ratio indicative for an exogenous administration of these agents, the major urinary metabolites of nandrolone (19-norandrosterone and 19-noretiocholanolone) reached levels above the WADA minimum required performance level (MRPL)(WADA TD2019MRPL) of 2 ng/mL for 48–144 h. As the daily dose recommended by the manufacturer is seven capsules, long-term usage of this product could not only be associated with AAFs in sports but also significant health risks.

In 2004, the results of a comprehensive study were published where 634 non-hormonal dietary supplements were purchased from 215 companies located in 15 different countries [42]. A total of 57 of these manufactures were also selling prohormones, and 45.6% of the tested products were obtained from these suppliers. The powders, tablets, fluids, and capsules were homogenized, extracted, derivatized, and finally analyzed by means of GC-MS. Out of the 634 tested products, 14.8% (=94) were found to contain AAS not declared on the label at concentrations between 0.01 and 190 µg/g. While 21.1% of the supplements bought from companies also selling prohormones were tested positive, 9.6% of the products obtained from the remaining suppliers contained AAS. An additional administration study demonstrated that an ingestion of the nandrolone prohormone 19-norandrostenedione at an absolute amount of 1 µg can result in an AAF concerning its metabolite 19-norandrosterone.

The study was repeated several years later and only 4 (=0.7%) of the 597 dietary supplements analyzed by means of GC-MS and LC-MS were found to contain unlabeled AAS, indicating that the prevalence of contaminated products has decreased since 2004 [48]. While the reason(s) for this phenomenon have not been proven, increased awareness and, consequently, improved production processes and/or supplement controls are likely aspects that contributed to the change in identified contaminations.

Shortly thereafter, the analysis of several vitamin and mineral tablets of a manufacturer also selling different prohormone products containing high amounts of unlabeled AAS, revealed the presence of metandienone and stanozolol at concentrations of 0.06–0.2 µg/tablet [49]. Again, it can be assumed that these cross-contaminations originate from using the same production line without proper cleaning. Even though the detected amounts were found to be too low to cause an AAF after the administration of one tablet, other factors such as a long-term application, varying concentrations of the contaminants, and metabolic differences between individuals could potentially lead to inadvertent doping cases.

In the same year, the Swiss anti-doping laboratory reported the findings of different steroids and/or prohormones such as testosterone, androstenedione, norandrostenedione, androstenediol, and DHEA in dietary supplements marketed as creatine and “mental enhancers” [50]. Only trace amounts of 45 ng–300 µg/capsule were detected, but a 3-day administration study with the creatine product containing 1.2 µg of norandrostenedione per capsule showed that the use of this product according to the manufacturer’s recommendations can result in the detection of the nandrolone metabolites 19-norandrosterone and 19-noretiocholanolone at concentrations close to the urinary MRPL of 2 ng/mL.

The analysis of 48 dietary supplements marketed as protein concentrates (*n* = 29), creatine preparations (*n* = 15), and “natural fat-burner” extracts from *Citrus aurantium* (*n* = 4) by means of 2D-GC-ToF-MS yielded two positive samples with prohibited AAS: A whey protein gainer was found to contain nandrolone (22 µg/kg), testosterone (70 µg/kg), and DHEA (63 µg/kg), and 5α-androstane-3,17-dione (398 µg/kg) and 19-norandrostenedione (304 µg/kg) were identified in a creatine product [51].

Probable cross-contaminations in the ng/g range with the prohormones 4-androstenedione and 19-norandrostenedione as well as testosterone, testosterone decanoate, and nandrolone decanoate were also detected in dietary supplements labeled to contain l-carnitine, different amino acids, proteins, and carbohydrates [52]

For some products, an intentional manipulation with pharmacologically relevant amounts (>1 mg/g) of unlabeled AAS was assumed [28,53]. Promised an increased strength and muscle growth, attributed to “new” ingredients with imaginary names [39].

For example, a high concentration of unlabeled metandienone was observed in several dietary supplements sold in the UK [53]. In one of these products, the detected amounts were found to vary significantly from capsule to capsule with maximum concentrations of 28.9 mg/g. An administration of these supplements according to the manufacturer’s instructions would result in supra-therapeutic doses of a steroid hormone, which has no clinical approval in Germany and several other countries. This would not only result in AAFs in sports, but also be associated with unforeseeable health risks, especially when used by women, children, and adolescents.

In March 2015, the doping control urine samples of 11 Bulgarian weightlifters training for the European Championships were found to contain the stanozolol metabolite 3′-hydroxystanozolol glucuronide [54]. Most of the athletes had declared the use of different supplements and/or non-prescription medications on their doping control form. After the AAFs were reported, all weightlifters as well as their coach stated to have administered a supplement called *Trybest* during training. The analysis of the product revealed the presence of unlabeled stanozolol at amounts of 1.7–4.2 µg per capsule, which can potentially result in the detected urinary concentrations of 3′-hydroxystanozolol. Different scenarios comprising supplement contamination, an intentional adulteration of the product by the manufacturer, and a deliberate sabotage were discussed, and eventually, the athletes were sanctioned as they should have been aware of the risk associated with the administration of dietary supplements.

SARMs are a novel class of anabolic agents, which are not only characterized by a high tissue selectivity and oral bioavailability, but also significantly reduced androgenic side effects [55]. Although no drug candidate has obtained clinical approval yet, different illegal products containing SARMS are available on the black market [56]. Moreover, the U.S. Anti-Doping Agency (USADA) has issued a warning that athletes are at risk of inadvertent doping with different SARMS and especially ostarine, which was found to be an unlabeled or misleadingly labeled ingredient of various dietary supplements and also present as contamination in such products [57]. Since 2017, the AAFs of several U.S. athletes could be linked to the use of such contaminated/adulterated dietary supplements, and reduced sanctions were therefore applied in all these cases [58,59,60,61,62,63,64].

#### 2.2.2. Stimulants

The category of stimulants commonly subsumes compounds that increase the activity of the central nervous system (CNS) and thus affect alertness, mood, appetite, and locomotion, as well as the sympathetic nervous system, resulting predominantly in cardiovascular effects [65,66]. They are one of the oldest classes of doping agents and, due to their transient effects, prohibited in-competition only. In the WADA Prohibited List [41], stimulants are divided into two categories: Specified stimulants such as e.g., methylphenidate and pseudoephedrine are widely available (e.g., in pharmaceutical products) and therefore more susceptible to inadvertent doping [65,66,67,68]. Consequently, the impending sanctions can potentially be reduced. By contrast, non-specified stimulants comprise strong stimulants as for example amphetamine. Stimulants are routinely identified in doping control samples by using GC-MS or LC-MS [65]. With the exemption of octopamine, the MRPL is set at 100 ng/mL for all stimulants considered as non-threshold substances. AAFs are however communicated only, when the reporting limit defined as 50% of the MRPL (i.e., 50 ng/mL) is exceeded [68]. Although sensitive detection methods are available since several years, stimulants are still popular among athletes [65]: In 2018, 15% of the reported AAFs accounted for these doping agents [40].

Stimulants have also been identified in numerous dietary supplements and, similar to AAS, both cross-contaminations and intentional admixtures have been described, the latter especially in products promoted for weight loss and energy improvement in order to rapidly obtain noticeable effects [6,65]. Additionally, stimulants naturally occurring in plant material can be problematic for athletes, in particular as the content can vary between species and various substance and plant names may exist.

Since 2004, athletes administering caffeine-containing products no longer risk an ADRV as the compound was removed from the WADA Prohibited List [65]. For the natural alkaloid ephedrine, a urinary threshold of 10 µg/mL applies [41], but nevertheless, careful considerations are in order when using *Ephedra sinica* preparations as some products were suspected to contain high amounts of ephedrine, arguably resulting from additions of the drug aiming to achieve significant performance-enhancing or weight-reducing effects [69]. The analysis of nine commercially available *Ephedra* products yielded a highly variable ephedrine content of 1–14 mg per capsule, which can be attributed to the use of different *Ephedra* species. But while natural *Ephedra* preparations usually contain several different alkaloids, two supplements appeared to be artificially fortified with synthetic ephedrine as it was the only detected stimulant (8 and 12 mg/capsule).

This also applies to other weight-loss supplements: In 2007, a Chinese herbal slimming tea and capsules were found to contain the synthetic drug sibutramine at concentrations of 1.8 mg/tea bag and 34 mg/capsule undeclared on the label [70]. Sibutramine is an amphetamine-derivative, which inhibits the re-uptake of the neurotransmitters serotonin and noradrenaline and is known to effectively suppress the appetite [6,70]. The detected amount of 34 mg is significantly higher than the doses administered in clinical studies (10–20 mg) and can therefore not only lead to AAFs in sports but also to unpredictable health risks, especially as the clinical approval of the drug was withdrawn in 2010 due to an increased occurrence of cardiovascular events.

Another stimulant often illegally added to dietary supplements marketed for weight loss and performance-enhancement is 1,3-dimethylamylamine (DMAA), also known as methylhexaneamine [71,72,73,74,75,76]. The drug is a synthetic aliphatic amine patented by Eli Lilly as nasal decongestant [72,73,74,75], but allegedly also a natural ingredient of the plant *Pelargonium graveolens* [77,78,79]. An extensive debate revolving around the study results published by Ping et al. [77] followed as follow-up studies returned conflicting results [72,73,74,75,76,78,79], and it cannot be excluded that dietary supplements prepared from *Pelargonium graveolens* extract, geranium oil, or geranium stem are artificially fortified with DMAA but labeled as “natural” products [71,72,73,74,75]. But also several entirely unlabeled dietary supplements were found to contain DMAA at concentrations of 136–415 g/kg [71].

The natural monoamine alkaloid phenylethylamine (PEA) and its synthetic derivatives function as neuromodulators in the CNS, resulting in stimulating effects similar to amphetamine [80,81,82]. Since 2015, these agents are found among the specified stimulants on the WADA Prohibited List [41,81]. PEA and related compounds are widely distributed as dietary supplements promising positive effects on energy and exercise duration [79,80]. Especially “natural” products containing material from the small tree *Acacia rigidula* were found to often contain phenylethylamines [82,83]. As the detected concentrations of PEA in some of these products (0.7–171.6 mg/g) were significantly higher than the natural levels of this compound in *Acacia rigidula* extracts (up to 1.5 µg/g), it can be assumed that also here admixtures of synthetic PEA to these products occurred. But as PEA is also produced by the human body, the differentiation of an illicit administration of the drug from endogenous levels is a complicated analytical task and requires the consideration of PEA metabolite profiles indicative for oral ingestion [81].

Besides PEA, also its derivative β-methylphenethylamine (BMPEA) is claimed to be a natural ingredient of *Acacia rigidula* [82]. However, this postulation was not confirmed in a study analyzing *Acacia rigidula* plant material for the presence of biogenic amines, which was initiated by the U.S. Food and Drug Administration (FDA) [83]. Nevertheless, BMPEA was identified in numerous dietary supplements advertised at metabolic activators and fat-burners at concentrations of 1–61 mg/g [82,83]. With estimated daily doses of up to 146 mg, the administration of such products could not only cause adverse effects but also inadvertent AAFs in sports [83].

In 2013 and 2014, the designer stimulant and PEA analog *N,N*-dimethyl-2-phenylpropan-1-amine (NN-DMPPA) was identified in the doping control urine samples of four athletes as well as a dietary supplement advertised as booster to increase motivation, strength, energy, and endurance, which was labeled to contain adrenergic amines from *Acacia rigidula* and caffeine [84]. The concentration was 122 µg/g, and BMPEA was also detected at an amount of 18 mg/g. The administration of a 3 g single-dose to three healthy volunteers (recommended daily dose by the manufacturer: 1 sachet containing 15 g of powder) resulted in urinary concentrations of more than 50 ng/mL (50% of the MRPL) for 22–23 h (NN-DMPPA) and 3–12 h (BMPEA) [85]. As the MRPL was installed to harmonize the analytical performance of the doping control laboratories and is not a threshold or detection limit, AAFs can also result from lower urinary concentrations [68].

Moreover, several cases of presumably unintentional doping with the PEA derivative *N*-ethyl-α-ethyl-phenylethylamine (ETH)/2-ethylamino-1-phenylbutane (EABP) have been reported, the occurrence of which can at least partially be attributed to inaccurate labeling of dietary supplements, obscuring the presence of this alkaloid [80,86] Different products were found to contain this designer agent at concentrations between 2 and 16 mg/g [80,86,87], and the administration of one of these products to three healthy volunteers resulted in urine levels higher than 50 ng/mL for 46–106 h [87].

In 2018, two AAFs with the specified stimulant heptaminol could be attributed to the use of fat-burners/pre-workout supplements labeled to contain 2-aminoisoheptane, which is an incorrect synonym for octodrine, a psychoactive stimulant of the CNS [88]. Following oral administration, the drug is metabolically converted to heptaminol, but as the misuse of both stimulants is prohibited in competition, these findings are predominantly relevant for an accurate results interpretation.

Furthermore, oxilofrine and the designer stimulant 1,3-dimethylbutylamine have been identified as adulterants in dietary supplements advertised as training boosters and slimming products [89].

#### 2.2.3. Other Substances

Although most of the reported cases on contaminated/faked supplements involve AAS or stimulants, there have been several findings with substances from other classes of doping agents.

In 2018, an athlete was repeatedly tested positive for the diuretic hydrochlorothiazide (HCTZ) [90]. Diuretics are drugs developed for the treatment of hypertension, and their misuse in sports is prohibited both in- and out-of-competition as they can not only interfere with the detection of other doping agents but also be misused to achieve rapid weight losses (relevant in sport disciplines with weight classes). In sports drug testing, they are routinely detected employing LC-MS, which yields urinary detection limits at the picogram level. In the athlete’s urine samples, low HCTZ concentrations of 8 and 13 ng/mL were observed, but the administration of any prohibited drug was vehemently denied. However, five different dietary supplements prepared in a compounding pharmacy were used during the period in question, and LC-MS analysis of four of these products revealed the presence of HCTZ at amounts of 2.1–4.6 ng/mL, 0–384 µg/capsule, and 0–147 µg/sachet. A subsequent administration study with three healthy volunteers demonstrated that the ingestion of HCTZ-contaminated powder (6.4 µg/g) can result in urinary HCTZ levels of up to 230 ng/mL, which supported an inadvertent administration of the drug by the athlete. Due to the sub-therapeutic and highly varying amounts of HCTZ detected in the different products, it was assumed that an accidental contamination during product manufacturing or packaging occurred.

Higenamine, or norcoclaurine, is an alkaloid acting as β_2_-agonist, whose misuse in sports is prohibited at all times [91,92,93]. Due to its natural occurrence in numerous plants such as *Annona squamosa*, *Aconitum carmichaelii*, *Plumula nelumbinis*, and *Nelumbo nucifera*, it is often found in pre-workout and fat-burner supplements. However, an unclear or missing labeling of the ingredients of such products has caused several cases of assumed inadvertent doping within the last years [91,92,93]. LC-MS analysis of different preparations neither listing higenamine or relevant plant extracts on their label yielded the alkaloid at concentrations of 0.02–14 mg/g. As the current reporting limit for urinary higenamine is 10 ng/mL [68], the use of such supplements could definitely cause AAFs in sports.

In 2009, also a peptidic compound called growth hormone releasing peptide 2 (GHRP-2) was detected in two different dietary supplements [94]. GHRP-2 and related peptides are agonists of the ghrelin receptor and thus stimulate the release of growth hormone (GH) from the pituitary. The respective tablets and drinking solution were bought in Cyprus and both correctly labeled to contain GHRP-2, however, the amino acid sequence and chemical structure provided with the tablets were incorrect. Even though the administration of these products cannot result in inadvertent doping in sports, it has to be expected that also unlabeled products contaminated or adulterated with GHRPs are sold on the supplement market. Moreover, the detection of GHRP-2 in such preparations is highly remarkable: Due to their physicochemical properties and enzymatic degradation in the gastrointestinal tract, protein- and peptide-based drugs have usually a poor oral bioavailability and are therefore administered by injection [95]. However, different GHRPs were found to have an unusual high oral activity [96]. Consequently, the administration of dietary supplements containing GHRP-2—Which had no clinical approval at the time of publication—At concentrations of 50 µg/tablet and especially 9 mg/ampoule can potentially result in pharmaceutical effects [94].

## 3. Contaminations of Drugs and Medical Preparations

Both pharmaceuticals and food are usually tested for the presence of contaminations and impurities at the part per million (ppm) level, which is sufficient to prevent any pharmacological effects, but it cannot rule out entirely implications for sports drug testing [34].

At the end of 2014, the diuretic HCTZ was detected in the in-competition urine sample of a Swiss athlete at an estimated concentration of 5 ng/mL [97]. The athlete had not declared the use of any dietary supplement, but the administration of several tablets containing ibuprofen, a non-steroidal anti-inflammatory drug (NSAID). Surprisingly, the analysis of the ingested analgesic as well as the respective retention sample provided by the manufacturer demonstrated the presence of HCTZ at a concentration of approximately 2 µg per tablet. According to the pharmaceutical company producing the NSAID, the contamination was located in the coating of the tablets and no indications could be found that the 10 ppm cleaning limit defined by current GMP guidelines was exceeded. In order to test the plausibility of the suspected scenario of inadvertent doping, two administration studies with placebo-tablets containing 2.5 µg of HCT were conducted and the collected post-administration samples were found to contain HCTZ at concentrations of up to 16 ng/mL. As these findings supported an accidental ingestion of the doping agent by the athlete, no sanction was imposed.

Another unexpected situation resulting in AAFs triggered by the administration of a permitted medication was published in 2015 [98]. Two athletes tested positive for the diuretic chlorazanil (0.3 and 1.3 ng/mL), an obsolete therapeutic never recorded in anti-doping statistics since the consideration of diuretics as doping agents in 1988. Both athletes denied the administration of the drug but declared the use of Malarone, a malaria chemoprophylaxis drug containing 100 mg of proguanil hydrochloride and 250 mg of atovaquone. While the analysis of the Malarone tablets did not reveal any contaminations with chlorazanil, additional experiments investigating a potential metabolic conversion of proguanil to the structurally related diuretic demonstrated that chlorazanil can be produced from the proguanil metabolite *N*-(4-chlorophenyl)-biguanide if elevated levels of formaldehyde—As it can occur in the course of creatine supplementation—Are present in the urine. Consequently, both AAFs did not proceed to ADRVs.

In contrast to these cross-contamination and unexpected bioconversion scenarios, also cases involving medical preparations intentionally fortified with unlabeled pharmaceuticals were discovered.

For instance, several allegedly herbal preparations were found to contain glucocorticoids such as hydrocortisone, betamethasone, and prednisolone, which were presumed as intentionally added to obtain a higher effectiveness of the therapeutics [99,100]. Glucocorticoids are steroid hormones with anti-inflammatory and immunosuppressive properties used for the treatment of various medical conditions [101]. In sports, their systemic administration is prohibited in-competition and the use of faked supplements could therefore not only cause adverse events but also ADRVs.

Insulin-like growth factor I (IGF-I) is an endogenous cytokine mediating the effects of human growth hormone (hGH), and the misuse of recombinant IGF-I and synthetic analogs in sports is therefore prohibited at all times [102]. In 2013, human IGF-I was detected in four dietary supplements containing deer antler velvet. Such preparations are frequently used in traditional Asian medicine, as the high content of growth factors promises various health benefits. While it remains debatable and certainly depends on the route of administration if any of the IGF-I is eventually bioavailable to the antler velvet consumer, the detection of deer IGF-I in athletes’ doping control samples would be reason for reporting an AAF.

Another particularly unusual case resulting in several AAFs with endogenous anabolic-androgenic steroids was reported during the FIFA Women World Cup 2011 [103]. Five members of a soccer team were tested positive after being treated with musk pod formulations. Musk pod extracts are widely used as traditional Asian medicine and known to contain various AAS whose administration in sports is prohibited [103,104]. Therefore, they have been included in “The list of medical products containing prohibited substances employed for doping” published by the State Food and Drug Administration of China. Consequently, sanctions between 14 and 18 months were imposed on the affected soccer players.

## 4. Food Contaminations

Besides dietary supplements and medical preparations, also food was found to be a potential source of inadvertent doping.

In several countries such as China and Mexico, the sympathomimetic and anabolic agent clenbuterol has been illegally used as growth promoter in animal production [105,106]. As a result, the edible meat is notably lean but was also found to be contaminated with clenbuterol residues, which can pose a health risk for the consumer and lead to AAFs in sports. Due to its anabolic and lipolytic effects, clenbuterol is listed among the anabolic agents in the WADA Prohibited List and is therefore prohibited both in- and out-of-competition [41,106,107]. In routine sports drug testing, clenbuterol can be detected in urine down to concentrations of a few pg/mL by using LC-MS approaches [106,107]. Until the amendment of Article 7.4 of the WADC in 2019, where the option to report atypical findings for clenbuterol if observed below 5 ng/mL of urine was introduced [1,108], no threshold applied for the detection of this drug in doping control samples, and even low concentrations resulted in AAFs and corresponding sanctions [107,109]. In an administration study with meat obtained from calves that were treated with clenbuterol at a dosage of 2 × 5 g/kg over a period of 37/43 days, the consumption by healthy volunteers resulted in urinary drug concentrations of up to 850 pg/mL in some of the participant’s urine samples [110].

Although the misuse of clenbuterol in food-producing animals is strictly regulated in most countries, several cases of clenbuterol intoxication following meat consumption have been reported from all over the world [105,107,111]. Symptoms can include tremors, tachycardia, palpitations, hypokalemia, nausea, headache, nervousness, dizziness, fever, chills, peripheral vasodilatation, and – in acute cases – breathing interruptions.

The extent of the clenbuterol problem in some countries was demonstrated by two studies published in 2012 and 2013 [106,109]: In 2011, the analysis of 28 urine samples collected from volunteers returning from or permanently living in China yielded a total of 22 (=79%) positive samples with clenbuterol concentrations between 1 and 51 pg/mL [106]. Moreover, the occurrence of five AAFs with the anabolic agent among athletes of the Mexican national soccer team induced a comprehensive investigation of urine and meat/food samples collected during the FIFA U-17 World Cup held 2011 in Mexico [109]. In 30% (=14/47) of the meat/food sample obtained from the restaurants catering the soccer teams, clenbuterol was detected at amounts of 0.06–11 µg/kg, and 52% (=109/208) of the doping control urine samples were found to contain the drug at concentrations of 1–1556 pg/mL. Due to the obvious problem of contaminated meat, none of the affected athletes were sanctioned.

However, the differentiation between an unintentional clenbuterol ingestion and doping still remains challenging. A promising approach represents the discrimination of clenbuterol enantiomers: While therapeutic clenbuterol is a racemic mixture of (+)- and (-)-enantiomers, animal tissue can be characterized by the enrichment of one of the stereoisomers [112,113]. While (+)-clenbuterol was found to be accumulated in pork and chicken tissue [112,113,114], the (-)-enantiomer was enriched in cattle and lamb meat [111,113]. Therefore, both the route of administration (pharmaceutical product vs. meat) and the type of ingested meat can potentially influence the ratio of clenbuterol enantiomers in human urine [115,116]. However, the enantiomeric ratio was not only found to vary depending on the analyzed tissue and species of meat-producing animals, but also on the withdrawal period before slaughtering [111,112,113], and more research on the excretion of clenbuterol enantiomers needs to be conducted before an approach adequate for routine application in sports drug testing is available.

Hair testing is also considered as an alternative strategy to discriminate clenbuterol misuse from contamination [117]. Due to its lipophilic properties, the drug binds permanently to the hair pigment melanin and the segmental analysis of hair can therefore provide valuable additional retrospective information on the time-point of clenbuterol ingestion.

In addition to clenbuterol, also other anabolic agents bear the potential to be misused as growth promoters in livestock production.

In a comprehensive administration study with 50 raw minced beef samples bought in different Belgian butcher shops, two of the participating volunteers were tested positive for the AAS nandrolone and clostebol [118]. As usually lower quality muscle tissue is used for the production of minced meat, it was assumed that the injection sites at the neck or tail base of the animals were processed into the consumed products.

After a Norwegian athlete was tested positive for the major urinary metabolite of the AAS metenolone, a comprehensive administration study was initiated in order to investigate the possibility of inadvertent doping caused by the ingestion of contaminated poultry [119]. For that purpose, chickens were either orally treated (1 mg/day over a period of 21 days) or injected (3 injections with 1 mg of a depot formulation on days 0, 7, and 14) with metenolone and slaughtered on day 22. Subsequently, the resulting meat was administered to eight healthy male volunteers and they were asked to collect urine samples for 24–48 h. GC-MS was employed both for screening and confirmation analysis. While the consumption of the meat obtained from orally treated chickens did not result in any findings with metenolone or its metabolite, half of the volunteers were tested positive for the parent compound 22–24 h following ingestion of the injected chickens. The metabolite could be confirmed in two samples collected 4–6 h post-administration. These findings demonstrate that also contaminated poultry can cause AAFs in sports, however, the respective athlete was still sanctioned as this scenario appeared very unlikely in his case.

Zeranol is a semi-synthetic non-steroidal growth promoter, whose misuse in sports is prohibited at all times [41,120]. Inadvertent doping with this drug can not only occur due to an illegal administration to meat-producing animals, but also due to the natural presence of structurally related mycotoxins in grains: Certain fungi species colonizing in wheat, maize, barley, and oats produce zearalenone, α-, and β-zearalenol, which can be enzymatically converted to zeranol after the consumption of contaminated cereals. As ADRVs with zeranol are very rare, the possibility of an accidental ingestion should be considered in case of AAFs in sports. Metabolic profiling was identified as a potential analytical strategy to distinguish an unintentional ingestion of the mycotoxins from zeranol doping.

A potential source for unintentional doping with the nandrolone metabolites 19-norandrosterone and 19-noretiocholanolone is the consumption of edible tissues (offal and meat) from non-castrated pigs/boars, which are naturally enriched with different steroid hormones [121,122]. After eating 310 g of a meal prepared from boar kidneys, heart, liver, and meat, the urine of three healthy male volunteers was found to contain 19-norandrosterone at maximum concentrations of 3.1–7.5 ng/mL for up to 24 h, which is above the urinary MRPL of 2 ng/mL [121]. The maximal values for 19-noretiocholanolone were 0.5–1.2 ng/mL. In sports drug testing, IRMS is routinely employed to demonstrate the exogenous origin of 19-norandrosterone detected in an athlete’s urine sample at low concentrations between 2.5 and 15 ng/mL [46,122]. As such urine levels would also be observed after the consumption of edible tissue from non-castrated pigs, another administration study was conducted in 2018, in order to clarify which impact the ingestion of boar offal has on the δ^13^C values of urinary 19-norandrosterone [122]. Two male healthy volunteers consumed a meal prepared from wild boar testicles and subsequently collected urine samples for a period of 24 h. Approximately 4 h following administration, maximum 19-norandrosterone concentrations of 4 and 8 ng/mL were detected employing GC-MS, and IRMS analysis yielded highly enriched δ^13^C values, which would constitute an AAF. Consequently, both athletes and anti-doping organizations should be aware of the risk associated with the consumption of boar products [46].

One of the oldest doping agents prohibited in-competition is the narcotic morphine [123]. For the urinary detection of this alkaloid, a threshold of 1 µg/mL applies in order to reduce the risk of inadvertent doping through the administration of pharmaceuticals containing codeine or the ingestion of poppy seeds [123,124]. However, a variety of studies demonstrated that the consumption of products containing poppy seeds can still cause AAFs in sports. In one study, eight poppy seed products commercially available in Germany were analyzed by means of GC-MS and the morphine content was found to vary from below 1 to 152 µg/g [123]. The seeds containing the highest amount of the alkaloid were subsequently used to prepare a poppy seed cake for an administration study including 9 healthy volunteers. Following ingestion, all participants were tested positive for several hours with urinary concentrations of up to 10 µg/mL. Similar results were obtained in a study published in 1990 [125]: While the consumption of 1–3 poppy seed rolls (containing 2 g of Australian seeds with a morphine content of 108 µg/g) did not result in urinary levels higher than 1 µg/mL, the ingestion of poppy seed cake (containing 15 g of Australian seeds with a morphine content of 169 µg/g) yielded concentrations of up to 2 µg/mL.

Due to the undeniable risk of inadvertent doping through the consumption of certain food and meat products, athletes are advised to take precautions and/or avoid certain meals. As there are currently no uniform international regulations or testing programs with regard to the presence of growth promoting agents in meat and the illegal use of such agents strongly varies between countries, this applies in particular to athletes traveling to international sports events [126,127].

## 5. Practical Aspects—Protection from Inadvertent Doping

The risk of inadvertent doping is predominantly connected to dietary supplements, which are aggressively marketed for muscle gain, fat loss, and boosting effects (mental enhancement). Therefore, athletes are advised to act with caution when intending the use such supplements [128].

If the use of dietary supplements is considered essential, acquiring supplements from low-risk sources is recommended. Information on vendor test results are available at e.g., the Cologne List (www.koelnerliste.com), the Informed Sport list in the UK (www.informed-sport.com), the NZVT list in the Netherlands (www.dopingautoriteit.nl/nzvt), etc.

In addition, dietary supplements produced by pharmaceutical companies are considered to exhibit low contamination risks as such products have not yet been reported as contaminated with doping substances [129].

In general dietary supplements should be considered carefully before use. A guidance for athletes and their advisers to minimize the risk of inadvertent doping is provided in the decision tree of the IOC consensus statement about dietary supplements and the high-performance athlete [8].

## 6. Conclusions

According to WADA’s principle of strict liability, every athlete is responsible for the presence of a prohibited substance or its markers/metabolites in his/her biological samples, irrespective of whether or not the ADRV was committed unintentionally or deliberately. Besides the use of dietary supplements and pharmaceuticals contaminated or artificially fortified with doping agents such as AAS, stimulants, and diuretics, also the consumption of food tainted with anabolic agents or naturally containing high amounts of prohibited substances can cause inadvertent AAFs in sports (summarized in Table 1). Whilst proof for the unequivocal causality between AAF and contaminated food or supplement ingestion is difficult to provide in most instances, plausibility beyond reasonable doubt was demonstrated in selected examples of the listed case studies. The most important strategy to protect athletes from these scenarios is an appropriate education. However, from a laboratory perspective, additional measures include the identification and implementation of novel long-term metabolites for exogenous AAS in order to improve both the retrospectivity and sensitivity of the detection methods, the usage of non-targeted approaches based on high resolution/high mass accuracy mass spectrometry to identify emerging doping agents, the provision of additional analytical data from administration studies, and the development of assays that contribute to a differentiation of an intentional administration from inadvertent doping.

## Figures and Tables

**Table 1:** Summary of findings. Various prohibited substances were detected as contaminants in dietary supplements, food products, or regular therapeutics that potentially or plausibly resulted in cases of adverse analytical findings.

Confirmed Sources of Prohibited Substances	Risk of Inadvertent Exposure with Prohibited Substance through	Case-Related Explanation Regarding Adverse Analytical Findings	Reference(s)
Dietary supplements contaminated with prohormones of nandrolone (e.g., 19-norandrostenedione)	Supplement consumption	n/a	[42,43,47,50]
Dietary supplement contaminated with prohormones of testosterone (e.g., 4-androstenedione)	Supplement consumption	n/a	[43]
Musk pod formulations naturally containing different anabolic-androgenic steroids	Treatment with traditional Asian medicine	yes	[103]
Meat contaminated with clenbuterol	Food intake	yes	[106,109,110]
		no	[111,116]
Meat contaminated with clostebol	Food intake	yes	[118]
Meat contaminated with nandrolone	Food intake	yes	[118]
Meat contaminated with metenolone	Food intake	yes	[119]
Offal and meat from non-castrated pigs/boars naturally enriched with different steroid hormones	Food intake	yes	[121,122]
Dietary supplement contaminated or adulterated with stanozolol	Supplement consumption	yes	[54]
Dietary supplements contaminated or adulterated with ostarine	Supplement consumption	yes	[58,59,60,61,62,63,64]
Dietary supplements contaminated with hydrochlorothiazide	Supplement consumption	yes	[90]
NSAID contaminated with hydrochlorothiazide	Administration of an analgesic	yes	[97]
Malaria chemoprophylaxis drug containing proguanil	*In vesica* conversion of proguanil metabolite	yes	[98]
Dietary supplement containing N,N-dimethyl-2-phenylpropan-1-amine & β-methylphenethylamine	Supplement consumption	no	[85]
Dietary supplement containing N-ethyl-α-ethyl-phenylethylamine	Supplement consumption	yes	[87]
Dietary supplement containing octodrine	Supplement consumption	no	[88]
Poppy seeds naturally containing high amounts of morphine	Food intake	no	[123,125]
